# Supplementary figures and images for: Comprehensive analysis of the functional impact of single nucleotide variants of human CHEK2
Source: PLoS Genet. 2024 Aug 15;20(8):e1011375. doi: 10.1371/journal.pgen.1011375 (PMC11349238; doi:10.1371/journal.pgen.1011375)

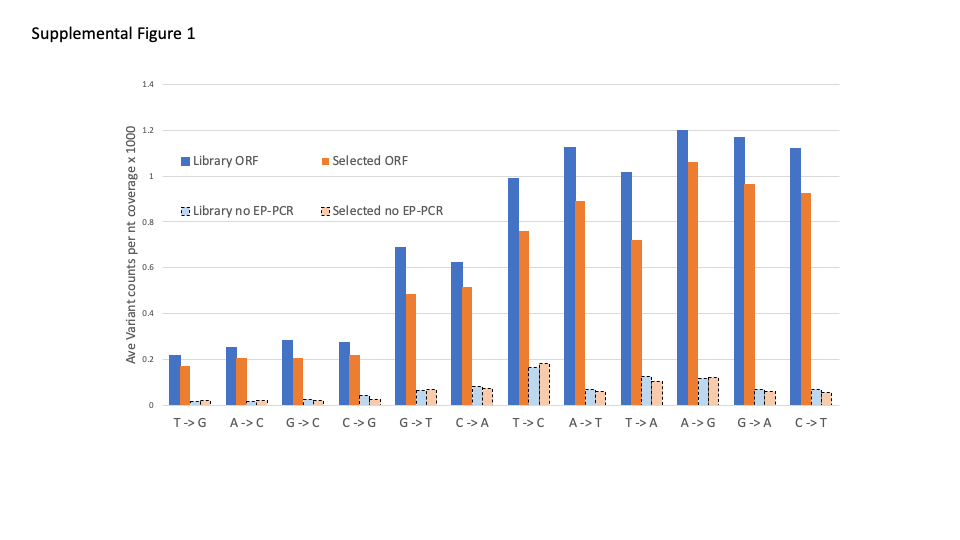

Supplement: S1 Fig — Variant frequencies are shown as the average of variant counts at each position divided by the sequencing coverage at that position x 1000. All frequencies were normalized to the most frequent wild-type nucleotide in the fragment being sequenced. Fragments include the 1629 bp mutagenized CHEK2 ORF (ORF) and two regions of the sequenced amplicon that were not mutagenized, combining 46 bp upstream of the CHEK2 ATG and 144 bp downstream of the CHEK2 stop codon (no-EPCR). For each, the results are shown for the library after introduction into yeast (Library), and after selection for rad53 complementation (Selected). (TIFF) [file pgen.1011375.s001.tiff]

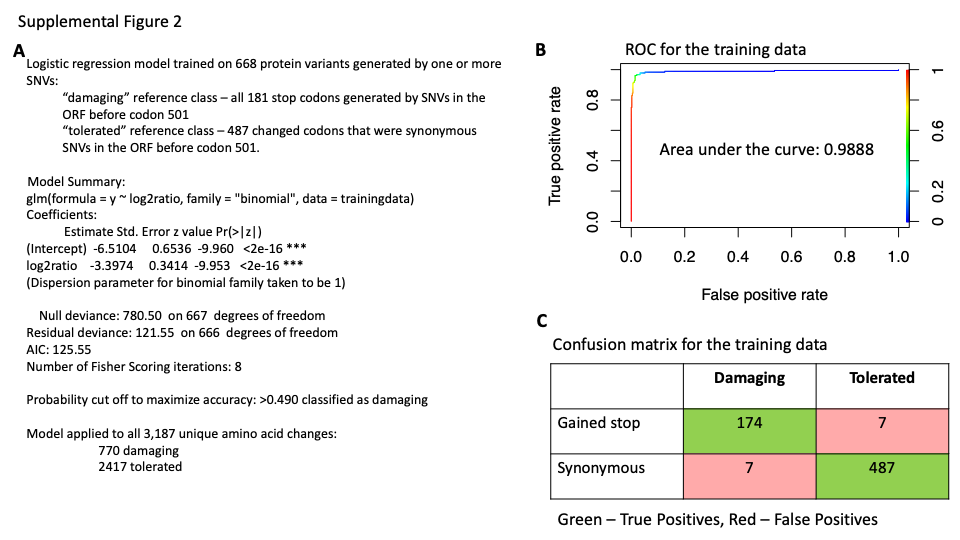

Supplement: S2 Fig — (A) Training data, model parameters, and output applied to the CHEK2 ORF. (B) Receiver operating characteristic (ROC) curve for the final model as applied to the training data. (C) Confusion matrix showing the numbers of training data (gained stops and synonymous SNVs) correctly classified (true positives, green) and incorrectly classified (false positives, red) by the model. Note that false positives in one class are equivalent to false negatives in the other class. (TIFF) [file pgen.1011375.s002.tiff]

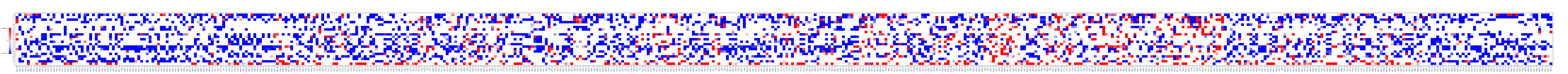

Supplement: S3 Fig — Residue positions and original amino acids are shown along the bottom while variants are listed at the left. Variants are colored according to their probability of being damaging (1.0 = red, 0.0 = blue), based on a logistic regression model of the screen depletion scores or RCS. (TIFF) [file pgen.1011375.s003.tiff]

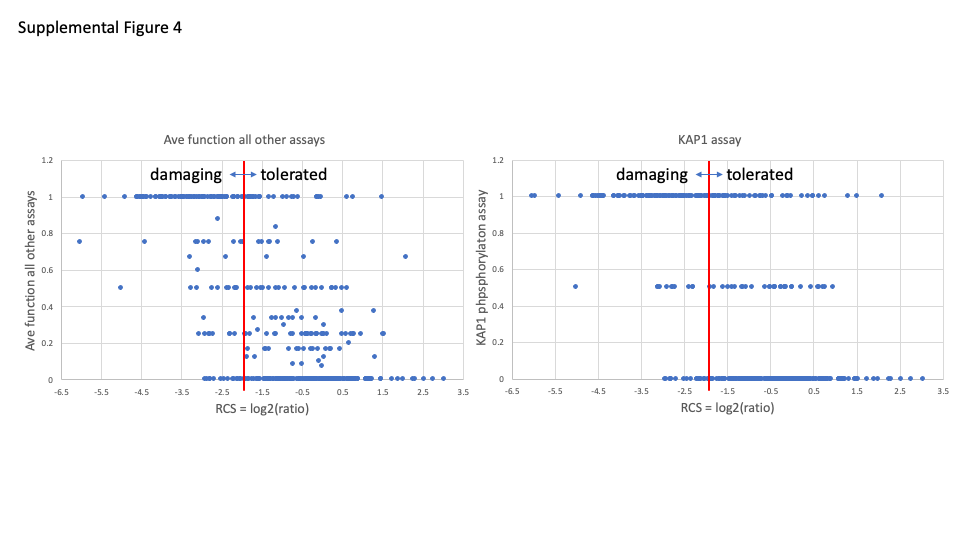

Supplement: S4 Fig — In both graphs the rad53 complementation score (RCS) score is on the X axis, and as a reminder a red vertical line shows the cutoff between damaging (< -1.9) and tolerated (> -1.9). On the y axis is the average score of other functional assays, where 1.0 is damaging, 0 is tolerated, and anything in between is intermediate (data in S3 Table). In both graphs, the variants with intermediate function in other assays are spread uniformly across the RCS scale. This is the case for the 443 variants that have been tested in at least one other functional assay (left graph) and for the subset of variants tested in the KAP1 kinase assay (by Stolarova et al. [22]). (TIFF) [file pgen.1011375.s004.tiff]

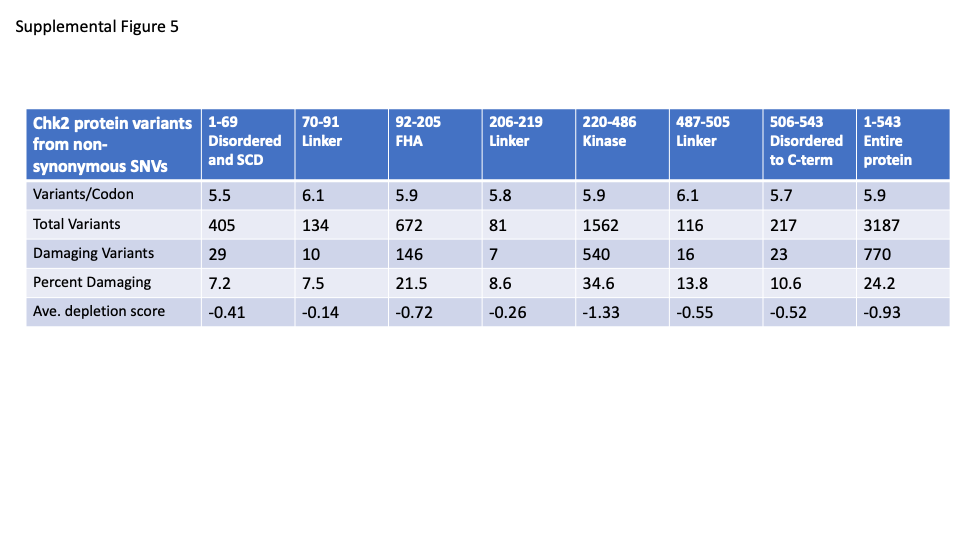

Supplement: S5 Fig — (TIFF) [file pgen.1011375.s005.tiff]

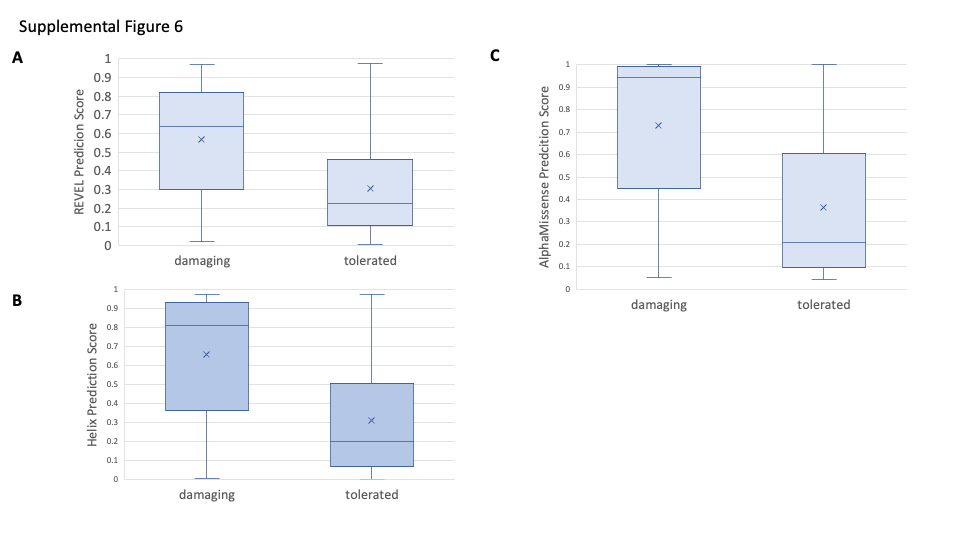

Supplement: S6 Fig — Comparison of functional classifications from this study to the prediction algorithms, REVEL [55] (A) and HELIX [23] (B), and Alphamissense[56] (C). In each, variants are scored on a scale of 0 to 1 (Y axis), with scores > = 0.5 predicting pathogenic (damaging) and scores <0.5 predicting benign (tolerated). The average REVEL, HELIX, and Alphamissense scores (x in the boxplot) for variants we found to be damaging was 0.566, 0.659, and 0.73 respectively, while the average scores for the tolerated variants was 0.304, 0.309, and 0.36 respectively. (TIFF) [file pgen.1011375.s006.tiff]

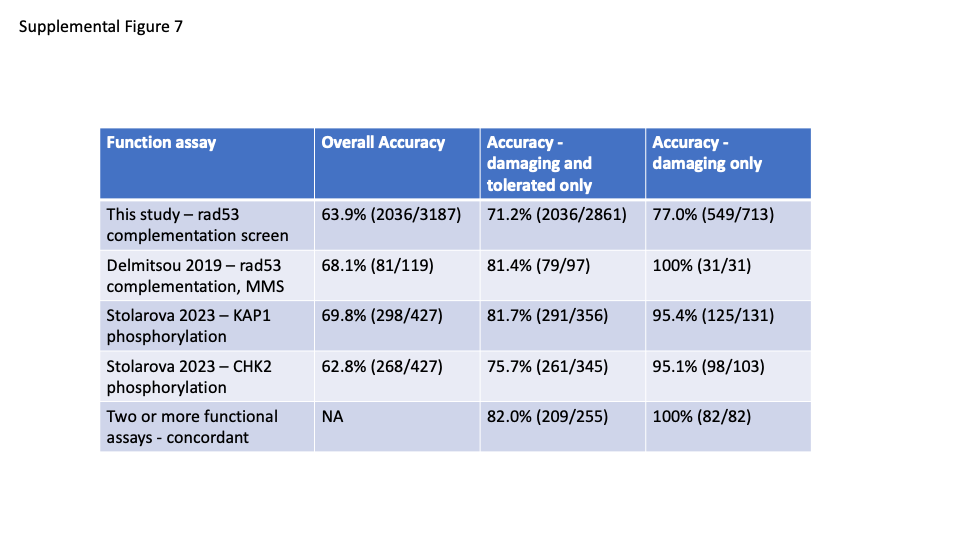

Supplement: S7 Fig — Overall accuracy: the fraction damaging, tolerated, and ambiguous or intermediate (if the assays included such a designation) that were accurately predicted by AlphaMissense. The “Accuracy of damaging or tolerated only” ignores all variants designated as ambiguous or intermediate by AlphaMissense or the functional assay. The last row includes only variants tested in more than one assay, where all assays agree that they are tolerated or damaging. (TIFF) [file pgen.1011375.s007.tiff]
