# Supplementary material for: Comprehensive analysis of the functional impact of single nucleotide variants of human CHEK2
Source: PLoS Genet. 2024 Aug 15;20(8):e1011375. doi: 10.1371/journal.pgen.1011375 (PMC11349238; doi:10.1371/journal.pgen.1011375)
Supplement: Attachment — Submitted filename: PLOS Genetics Response to Reviewers.docx [file pgen.1011375.s011.docx]

PLOS Genetics Reviewer Comments

**Reviewer #1:** The report by McCarthy-Leo et al is a substantial contribution to our understanding of the functional impact of SNV’s in CHEK2. The paper is detailed and pays considerable attention to considering the new data in the context of previous functional screens and application to clinical variant classification.

The authors present a model that categories variants, based on their RCS, as damaging or tolerated (which is supported by their data). However, comparison to previous reports that include a third intermediate category is difficult and this comparison is an important aspect of this report and its anticipated utility for variant classification. Have the authors tried to achieve a model with an intermediate category? If so, what was the outcome?

The short answer to the reviewer’s question is: Yes we have tried to implement an intermediate category and we found that it ***decreases*** overall accuracy when benchmarked against other functional assays or VEPs.

Unfortunately, the data from our screen do not support the use of an intermediate functional category, as detailed below. The RCS scores could not be shown to correlate with a third, intermediate functional category. Nevertheless, we have included the raw RCS scores and the logistic regression probability scores for all variants because these may prove useful when combining with future functional data to develop increasingly accurate functional and clinical classifiers.

Our model for calling functionally damaging and tolerated variants is based on the use of high-quality training data (nonsense and synonymous variants) for those two classes. There is no comparable high-quality training data available for intermediate function (as discussed further below). Nevertheless, we could use a heuristic approach to try to find appropriate cutoffs in our screen score (RCS) that would flank the intermediate category and improve overlap with other assays that include an intermediate category. The two plots below illustrate how this approach would not improve the overlap with these other datasets. Both graphs have our RCS score on the X axis, and as a reminder a red vertical line at our cutoff between damaging (< -1.9) and tolerated (> -1.9). On the y axis is the average score of other functional assays, where 1.0 is damaging, 0 is tolerated, and anything in between is intermediate. One can see that the variants with intermediate function in other assays are spread uniformly across the RCS scale. Importantly, it is evident that any vertical line that one might draw to capture some of the intermediate values would necessarily eliminate *more* of the concordant values (1 or 0). This is the case for the 443 variants that have been tested in at least one other functional assay (left graph) and for the subset of variants tested in the KAP1 kinase assay (by Stolarova et al.).

It is also worth emphasizing again that the intermediate classification in other functional studies appears to be the least reliable classification. As we point out in our paper, most of the disagreements between assays are due to the intermediate calls. For example, of the 108 variants scored as intermediate in a least one of the other 14 functional assays and with at least one other assay to compare with (not including our assay), **94% (101/108) of the intermediate calls *disagree* with another assay**. The other assays were in agreement on the intermediate call for only 7 variants. One of these (I448S), was found to be intermediate in 4 out of 4 other assays (and we discuss this variant in the paper), while the other six were only assayed and agreed upon between the KAP1 and CHK2 phosphorylation assays. Moreover, while the KAP1 and CHEK2 phosphorylation assays agreed on 6 intermediate calls, they disagreed on the remaining 71 variants where one or the other assay called intermediate function: these account for 79% (71/90) of the disagreements between those two assays.

I have a small number of other minor comments and suggestions.

1. “Patient screening” might be better expressed as “susceptibility screening”

We agree with this suggestion and have made the change in the abstract and introduction.

2. It could be made clearer that “patient samples” are blood samples not tumour samples.

We have clarified that we are talking about germline (inherited) variants and not somatic variants or tumor samples.

3. “Cancer-causing” would be better expressed as “associated with cancer risk”

This change is reflected in the revised manuscript.

4. In many instances the term “mutation” could be replaced with pathogenic and likely pathogenic variants.

We changed “mutation” to “variant” in all appropriate instances.

5. In results, first para, ; “all mutations” – are the authors referring to all possible nucleotide substitutions? …as the para goes on to describe that two SNVs were not identified in the sequenced library.

We have replaced this with “all transitions and transversions are represented” to clarify in that sentence that we are referring to types of nucleotide changes created by error prone PCR.

6. “We found that most of the individual variants that did not fully complement rad53 were depleted in the screen (ave. RCS -3.64, SD=1.12), while most of the individual variants that fully complemented RAD53 were not depleted…”. Please provide n for “most”.

We have replaced the word “most” with an *n* for each set being compared in the individual *rad53* complementation screen. This also makes clearer that we are not comparing the numbers of variants depleted (RCS <0) or not depleted (RCS>0). Instead, we are comparing the ave RCS for the fully complementing and the non-complementing set.

7. Please clarify “The also data provide…”

We changed that to, “The data also provide…”

8. ENIGMA does not themselves have patients - please edit.

We have removed “patient” from the discussion regarding the ENIGMA data.

9. “Conferred by” would be better expressed as “associated with”

This change has been addressed in the revised manuscript.

**Reviewer #2:** In this manuscript, McCarthy-Leo et al. first reinvented the yeast RAD53 complementation assay, which they then used to evaluate all possible SNVs (-1) affecting the ORF in the human CHEK2 gene. The variants were generated using a well-balanced EP-PCR, and the PCR products were used to prepare constructs for yeast transformation and subsequent RAD53 complementation assay in the presence of 5-FOA. Next, they determined the abundance of individual SNVs by NGS before and after the complementation assay, which allowed them to discriminate between complementing SNVs (not depleted, retaining CHK2 kinase function) and non-complementing SNVs (depleted, indicating SNVs with impaired CHK2 function). Finally, the authors demonstrate the consistency of their results across the CHEK2 ORF and provide evidence for the correlation of their results with CHK2 function, ClinVar data, and data from previous analyses.
Overall, the data presented in this manuscript are of high quality and represent an original contribution, as no other AVE CHEK2 data have been published. Although the assay involves the yeast model and thus (as correctly discussed by the authors) pursues RAD53 complementation (which is not equivalent to the natural CHK2 function in human cells), this study will be of interest to many researchers from a broad spectrum of theoretical, preclinical, and clinical research. The work is technically robust and well suited for publication.

Thank you for your review. We have addressed your suggestions below in the manuscript.

Minor points:
The authors provide some initial evidence of their assay using a set of 30 manually constructed variants (Fig. 1); however, 29 variants are shown in this figure.

We thank the reviewer for catching this error. One variant (A17S) was inadvertently left off the figure and has now been added.

Forkhead domain (FHA) should be described as forkhead-associated domain.

We have corrected all instances to forkhead-associated (FHA) domain or FHA domain.

Regarding the comparison with the ENIGMA consortium study, the numbers in this section should be revised as 430 VUS were successfully analyzed by both functional assays in the ENIGMA study (not 427); moreover, the results in this manuscript are compared with 427 variants in the KAP1 assay, but with 327 variants in the CHK2 assay.

When comparing our data to the ENIGMA data we only compared missense variants that were SNVs (thus excluding c.349_350AG>AC/p.Arg117Ala) and those that were designated by the authors as functionally WT-like, functionally intermediate, or functionally impaired. Two variants (p.Arg521Gln and p.Arg521Trp) were not given one of those designations because the authors found them to be WT-like in the kinase assays, but not sufficiently nuclear localized. Therefore, ultimately, we could only compare our results with 427 of the 430 variants assessed by Stolarova et al. We edited the text to make this a little clearer. Also, there was a typo in referring to the number of variants from the CHK2 phosphorylation assay, which should be 427. We corrected that to read “..318/427 (74.5%)” were in agreement with our data.

Authors should note in the Discussion section that the discordance between individual functional screens may also be caused by differences in the model organisms used for the functional assays (the current discussion focuses more on the comparison of different readouts in yeast studies). Whether this phenomenon could explain the discordant results between this manuscript and ENIGMA studies regarding NLS would be worth commenting, as all nonsense variants from S500 were found to have normal activity, in comparison to ENIGMA study, where all tested truncating variants up to (including) p.523fsX had impaired function due to aberrant localization (see Figure 3B in Stolarova et al., reference 26).
Finally, it should also be noted that for variants affecting pre-mRNA splicing, the results of functional synthetic assays may be very different from the biological reality in situ.

We appreciate the feedback regarding the discordance between functional screens. The yeast model does have its limitations. We have expanded on this and included more discussion of the C-terminal 43 residues in the second to last paragraph of the revised Discussion.

**Reviewer #3:** This study presents a deep mutational scan of nearly all possible single nucleotide substitutions in CHEK2 using a yeast-based complementation assay. These kinds of high-throughput experimental assays are becoming increasing important for variant analysis, with potential clinical implications. I do think the work is technically sound and potentially very useful to the community, and I believe it should be published in PLOS Genetics. However, there are a few issues that I think should first be addressed.

The paper positions itself well within the context of past work in the CHEK2 field, but there is little discussion of other work in the DMS/MAVE field. These high-throughput assays have become increasingly common in recent years, including several other yeast complementation DMS experiments, so it would be good to mention them and discuss how this compares. I would make sure to include the term DMS or MAVE in the abstract, to make it more discoverable.

Thank you for the suggestion. We added DMS to the abstract and expanded citations to the DMS and MAVE approaches and examples in the last paragraph of the introduction.

I would love to see a variant effect map of amino acid substitutions (ie heat map with all missense variants colored by effect), which is typically included in most DMS studies. I find these to be more interpretable and useful than just showing the averaged variant effect per position.

We agree that variant effect maps (VEMs) are an excellent way to summarize and visualize the data. We have added one VEM to the paper (Supplemental Fig 3). The VEM depicts amino acid substitutions and uses the probability values from logistic regression as the scores, which makes for a good visual summary of our functional classifications. As suggested below, we also intend to submit our raw data to MAVEdb, which will enable generation of VEMs from various scores in our data.

This dataset is presented as being useful for screening VUS, but from the paper, it's difficult to assess how useful it actually is. Actually, the agreement with previous lower throughput assays doesn't seem great. Since really what people are interested in is performance on missense variants. It would be helpful to show a typic ROC AUC analysis on the known (likely) pathogenic vs (likely) benign missense variants from ClinVar. I know there aren't a lot, but it should still be performative, and then this could also be compared to the computational predictors. Note that even if this analysis shows that the experimental measurements are 'worse' than the computational methods, in my opinion that doesn't take away from the fact that this study is important and should be published because 1) DMS data represents independent information, 2) it is important for us to learn how well these types of assays perform using different genes and different experimental methodologies, and 3) many of the computational predictors are trained on human variants so their performance can be overstated (the exception being pure evolution-based methods).

We maintain that the usefulness of our dataset is in providing an additional datapoint for each variant that can be used in future multifactorial models to predict pathogenicity. Our datapoint is one measure of function, or more precisely, the probability of a variant being damaging to function based on our experimental data. We understand the reviewer’s concern that “the agreement with previous lower throughput assays doesn't seem great.” We realize that it is not comforting to hear, but we do point out that the other assays often do not agree with each other to the same extent. For example, of 428 variants tested in more than one functional assay (not counting ours), all assays agree on only 297 (69%); adding our data to that analysis, all assays agree on 260 (60%). In the revised discussion, we offer some possible explanations for the disagreements and further suggest that the solution to this problem is to combine functional data to derive a higher confidence set of annotations.

Regarding comparisons to ClinVar classifications, as the reviewer suspected, there are only 9 missense variants annotated as pathogenic or likely pathogenic and only 5 missense variants annotated as benign or likely benign. 14 is not a very useful number for training or making AUC comparisons with the computational predictors. Another problem is that some ClinVar classifications depend on functional assays or function prediction, making any potential comparisons somewhat circular. Nevertheless, we added the ClinVar classifications for the missense variants to our table comparing all of the functional assays (Supplemental table 3). We also discuss in the paper the subset of these 14 variants where the functional assays appear to disagree with each other or with our data, including for R117G, G167R, S428F, and I448S.

Is it possible to provide any confidence/error estimates for the RCS scores? For example, I imagine library coverage is not perfectly uniform, so variants that have lower coverage in the library should have lower confidence. A lot of studies don't provide complete coverage due to such issues, so I can imagine some lower quality measurements were included to make the dataset almost complete. If you select a higher confidence, is the agreement with previous assays or ClinVar labels better?

We added P-values for the RCS score (Supp Table 1) - based on significance testing of the difference between SNV frequency in the library before and after selection - though we found that this does not provide a confidence estimate in the predictive power of each RCS score. More importantly, we added a probability score for each variant (Supp Table 2 and Supp Fig 3) based on the logit model trained on gained stops (presumed damaging) and synonymous variants (presumed tolerated). This score is directly interpretable as the probability that a variant is damaging, based on the RCS score. While the variant counts in the library do vary over a broad range, we find that the predictive power of the classifier is the same all along the range. For example, we find that predictions for the least abundant 5% are as accurate as the they are for the remaining 95%, and that the rate of agreement with other assays is also robust over the full range of library counts.

The RCS scores should be deposited in MaveDB (www.mavedb.org) to improve accessibility.

Thank you for this suggestion. We will initiate submission of our data to MaveDB once our paper is accepted for publication and we will add that source to the paper’s comment on data availability.

**Reviewer #4:** The authors have provided a MAVE analysis of all CHEK2 single nucleotide variants using a high throughout growth assay in rad53 deficient yeast. The study is attractive because it goes well beyond all previous CHEK2 functional studies by evaluating all possible SNVs. The focus on growth rather than DNA repair is a useful addition to the compendium of assays now available for CHEK2 variants. However, the problem with this and other assays is that there is a high proportion of discrepant results. Perhaps as suggested by Boonen et al, and echoed by these authors, consistency in 2 or more high quality assays will be needed.

While interesting, there are a number of significant issues that must be addressed.

The biggest problem is that the analysis is limited to a binary cutpoint, which yields damaging and tolerant categories. The problem is that function is very clearly a continuous measure and studies of CHEK2 and other genes have shown that intermediate functional effects with associated intermediate risks of disease very clearly exist. The authors must recalibrate their assay and apply a two-component mixture model based on damaging and tolerant standards (perhaps nonsense and synonymous variants). The VarCall model that has been used for BRCA1 (Clark et al 2022) and BRCA2 could prove effective. Application of this model may help to resolve the high degree of discrepancy (25%) that the authors report for the KAP1 phosphorylation assay that is currently considered the standard for CHEK2 variant evaluation.

We agree that there are likely to be CHEK2 variants with intermediate function and that it will be interesting (though very difficult) to eventually see whether intermediate function corresponds to intermediate risk. Unfortunately, the data from our screen do not support the use of an intermediate functional category, as detailed in our response to Reviewer #1’s similar comment. The RCS score could not be shown to correlate with a third, intermediate functional category. Nevertheless, we have included the raw RCS scores and error and the logistic regression probability scores for all variants because these may prove useful when combining with future functional data to develop increasingly accurate functional and clinical classifiers.

We recognize that the variant calls between our model and Stolarova et al. are discordant for around 25% of the variants. However, within the Stolarova et al. study itself, the two assays (KAP1 phosphorylation and CHK2 autophosphorylation) were discordant in around 21% of the variants they studied. Moreover, there are similar levels of disagreement among many of the functional assays, as we mention in the manuscript. This highlights the need to combine data to support variant functional predictions.

Supplementary Figure 2 should be a main figure in the manuscript but it must be improved to show the distribution of the stop codons, synonymous and missense variants. The current Figure 2 is insufficient for this purpose.

We added a confusion matrix to Supp Fig 2 to show the distribution of true and false positives among the damaging and tolerated predictions of the model. Supp Fig 2 cannot show information about missense mutants. Supp Fig 2 shows the logistic regression model and an ROC curve based only on the training data. An ROC based on training data is not normally considered a significant finding – it is just what is expected. We agree, however, that it is useful to show how well the model separately predicted each training class (nonsense and synonymous) so we added the confusion matrix.

The discrepancies for nonsense and synonymous standards and for previously characterized variants is considerable. In the absence of a method that provides probability of pathogenicity for each variant, and allows clear assessment of each variant, it is not clear how the presented data can be utilized to inform the clinical classification of any CHEK2 variants.

The accuracy for calling nonsense and synonymous variants is actually pretty good as these assays go (97.9%), but we get the point that our assay is not perfect. We would argue that none of the functional assays are perfect, and this is supported by their poor overlap with each other, with our data, and with VEPs. Because of this, a central conclusion of our paper is that functional data needs to be combined to derive high confidence assignments of function for each variant. Our screen provides one data point for each variant. Moreover, the ACMG guidelines use functional studies as only one piece of evidence to support sequence variant classification. The focus of our functional study was to assess how well Rad53 complementation predicts CHK2 function. This work is to provide functional evidence to be used in combination with other CHK2 functional study results as well as other evidence such as allele frequency, segregation data, and family history. These data are weighted together and applied to the clinical classification guidelines. We have expanded on these points in the second paragraph of the Introduction and last paragraph of the Discussion.

The authors combine or merge SNVs within single amino acids by combining or averaging scores. By their own admittance, there are nonsense variants that yield tolerant activity and there are likely synonymous variants that yield damaging activity. There are also potential effects on RNA splicing (which is not measured or predicted here) and on RNA stability. For this reason the standard in the field is moving towards analysis of individual SNVs rather than amino acids. The authors should re-evaluate their data at the SNV level with this in mind.

We agree that it will be important to eventually determine the functional impact of individual SNVs. Because our assay uses only the CHEK2 ORF and is performed in yeast, it is likely to miss many non-coding effects. Nevertheless, to facilitate future analyses, we included the SNV level data. We also added a comment to the Discussion acknowledging that non-coding effects (like splicing) might explain some of the differences between assays performed in yeast and mammalian cells. However, our analysis focused on predicting the functional impact of amino acid changes. To this end, combining data from multiple SNVs that lead to the same coding change filtered out some noise and potential non-coding effects and improved accuracy and overlap with other functional assays. For example, using multiple SNVs to score amino acid changes improved accuracy of damaging calls from 87.2% (206/236) to 91.9% (217/236), and improved the accuracy of synonymous calls from 94.2% (1019/1081) to 99.4% (1074/1081).

Statements reporting “significantly more depleted” are not detailed enough. Numbers of variants with discrepancies should be shown.

Thank you for that suggestion. In the text references to Fig 2, we added the actual RCS averages and SD to make clear that we are comparing the average RCS of groups. That sentence now reads: “…the pathogenic variants were significantly more depleted (mean RCS -3.15, stdev=1.49) than the VUS (mean RCS -1.10, stdev=1.54) or the variants with conflicting annotations (Fig 2A).” The *n* for each group is shown in the figure. The p-values for the differences in means are in the figure legend, while the box and whisker plots show the distribution of values and outliners.

Reporting of the correlations with VEPs, such as AlphaMissense, needs to include measures of sensitivity and specificity.

We have mentioned the accuracy of VEPs in the text and shown the accuracy of the Alphamissense predictions for various experimental datasets in what is now Supp Fig.6. The accuracy = true positives/(true positives + false negatives). In this binary classification problem, the number of false negatives equals the number of false positives, so accuracy is a measure of both sensitivity and specificity.

The report that the last 43 amino acids are not important for function of CHEK2 is interesting. However, the authors then show that 10.6% of variants in the C-terminus are damaging. This is confusing. Also, it is not clear if the C-terminus is considered in the reporting of missense variant effects. A clear explanation of the relevance of the terminal 43 amino acids for all aspects of the analysis is needed.

While the functional classifier used gained stops and synonymous variants from amino acids 1-500 as training data, we applied the resulting model to the entire protein, including all missense variants. We have expanded the Discussion section (second-to-last paragraph) to further address and highlight apparent discrepancy between assays in mammalian cells and yeast regarding the C-terminal 43 amino acids. We also point out that we do not have a good explanation for our finding that 25 out 249 missense variants in this region appear to be damaging in the yeast assay. The best we can offer is that more assays need to be done for these variants, both in yeast and mammalian cells.

It is not clear how the authors account for possible temperature sensitivity effects on protein stability caused by individual variants in the yeast model.

It is possible that temperature sensitive variants may have different thermostabilities in yeast and human cells because of the different optimal growing temperatures of these organisms. (A nice example is pBRCA1 R1699W which is a functional transcription activator at 30 deg C but not 37 deg C in both yeast and human cells. The mutant is functional at the normal temperature for yeast but not at the normal temperature for human – Worley et al., Cancer Biol Therapy. 2002 Sep-Oct;1(5):497-501). We have no way of testing or controlling for these possible differences in our assay, but we do point out in the Discussion that differential thermostability is one way that variants could behave differently in yeast (second to last paragraph of Discussion).

The authors missed an opportunity to emphasize the clinical utility of their findings. Strangely, results from case-control associations studies for breast cancer are mentioned at the end of the Discussion but no details are provided in Results or Methods. This must be corrected.

We thank the reviewer for this suggestion. We tried to stress in the manuscript that we are focused on determining the functional impact of CHEK2 variants. We mention the case-control studies from the ENIGMA project in the context of overlap with our data. We depict the overlap in Supplemental Table 3 and we cite the ENIGMA burden analysis (not our own analysis), which supports the idea that overlapping functional data is more reliable for predicting clinical risk.

It is absolutely essential that correct terminology be used throughout the manuscript. “Classification” is a term reserved for clinical classification or determination of the clinical relevance of a variant. Functional studies do not “classify” variants. Functional evaluation and “categorization” of variants should be used in this manuscript in the absence of any information on clinical relevance.

We use ‘classify’ because we used a machine learning algorithm (also known as a classifier) to place each variant into a functional class (damaging or tolerated) based on our experimental data. However, we agree that we should do better at distinguishing between clinical classification (e.g., pathogenic vs benign) and functional classification. To make this clearer and to avoid confusion with clinical classification, we have edited the manuscript so that now we state in every case which classification system we are referring to. For example, we now say we “functionally classified variants…”, or we “classified variants as either tolerated or damaging to CHEK2 function…”; in contrast, when we are referring to (usually other people’s work) a clinical classification, we say, “clinical classification as pathogenic or benign…” Finally, we note that several authors in the CHEK2 variant characterization field also refer to ‘functional classification’ as distinct from clinical classification (e.g., Bell 2007, Crisanthar 2008, Boonen 2022, Kleiblova 2019, and Stolarova 2023).
