# Supplementary material for: Comprehensive analysis of the functional impact of single nucleotide variants of human CHEK2
Source: PLoS Genet. 2024 Aug 15;20(8):e1011375. doi: 10.1371/journal.pgen.1011375 (PMC11349238; doi:10.1371/journal.pgen.1011375)
Supplement: Attachment — Submitted filename: Response to Reviewers comments 2.docx [file pgen.1011375.s012.docx]

Response to Reviewers comments on McCarthy-Leo et al. (PGENETICS-D-24-00029R1).

Reviewer #1: Thank you for your careful and considered response to my comments, questions and suggestions.
Could the authors be more explicate about their data not supporting the use of intermediate functional category in the text of the manuscript? The spread of variants called intermediate in other assays across the RCS scale (as illustrated in the figure provided in the response), helps to further understand the nature of the data and why a third category is not proposed by the authors.

We have added a second paragraph to the Results section, “*Classification of Non-Synonymous SNVs as functionally Damaging or Tolerated”,* to explain why our RCS could not be used to classify variants as having intermediate function and we included the figure from our previous response as Supplemental Fig 4. In the same paragraph, we also begin to point out that the intermediate classification appears to be unreliable across other assays. This previews a point that we detailed later in the paper. The raw data (numbers) for Supplemental Fig 4 were already in Supplemental Table 3.

We also added a comment to the last paragraph of the results section, “*Functional Consequences of Missense Variants”,* to emphasize that most of the disagreement between the Stolarova KAP1 and CHK2 phosphorylation assays is among variants that one of the assays called intermediate in function. This was another comment we had mentioned in our previous response and it now seems worthy of being in the paper given the increased emphasis on explaining the intermediate category.

Reviewer #2: The authors have adequately addressed my comments. This manuscript will be of great interest to many researchers/clinicians in the field of oncogenetics.

Thank you. Please see responses to Reveiwer#1 and #2 for additional edits to the manuscript.

Reviewer #3: I thank the authors for their careful consideration of my comments, and I now think this paper is ready for acceptance. However, I would suggest it is very important that the variant scores first be deposited in MaveDB so that the accession number can be included in the paper. You can do this and set your dataset to private, changing it to public after acceptance.

Thank you for the suggestion. We have deposited the data in MAVEdb and edited a sentence in the Results section to read:

“These results are summarized in an expandable variant effect map (Supplemental Fig 3) and are deposited into MAVdb [45] (accession urn:mavedb:00001203).”

Note: There appears to be no way to obtain a permanent accession number without making the data public. Our data, therefore, is now publicly available at MAVEdb.
